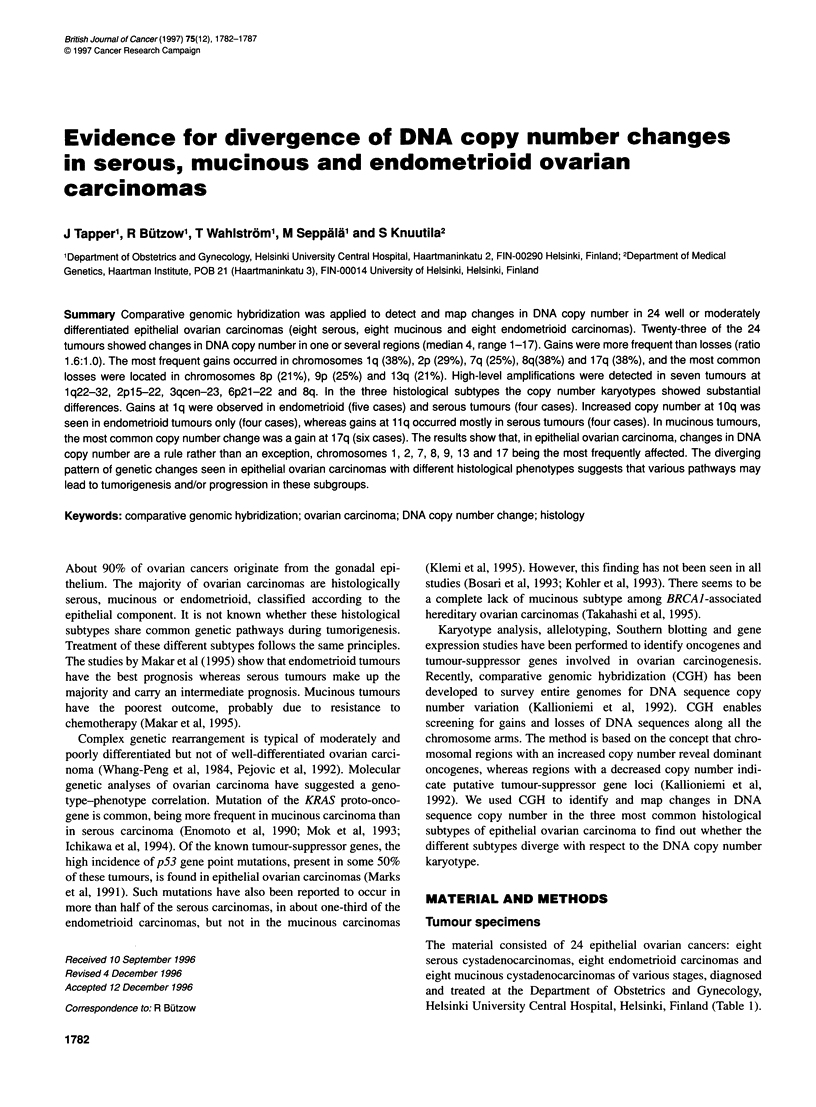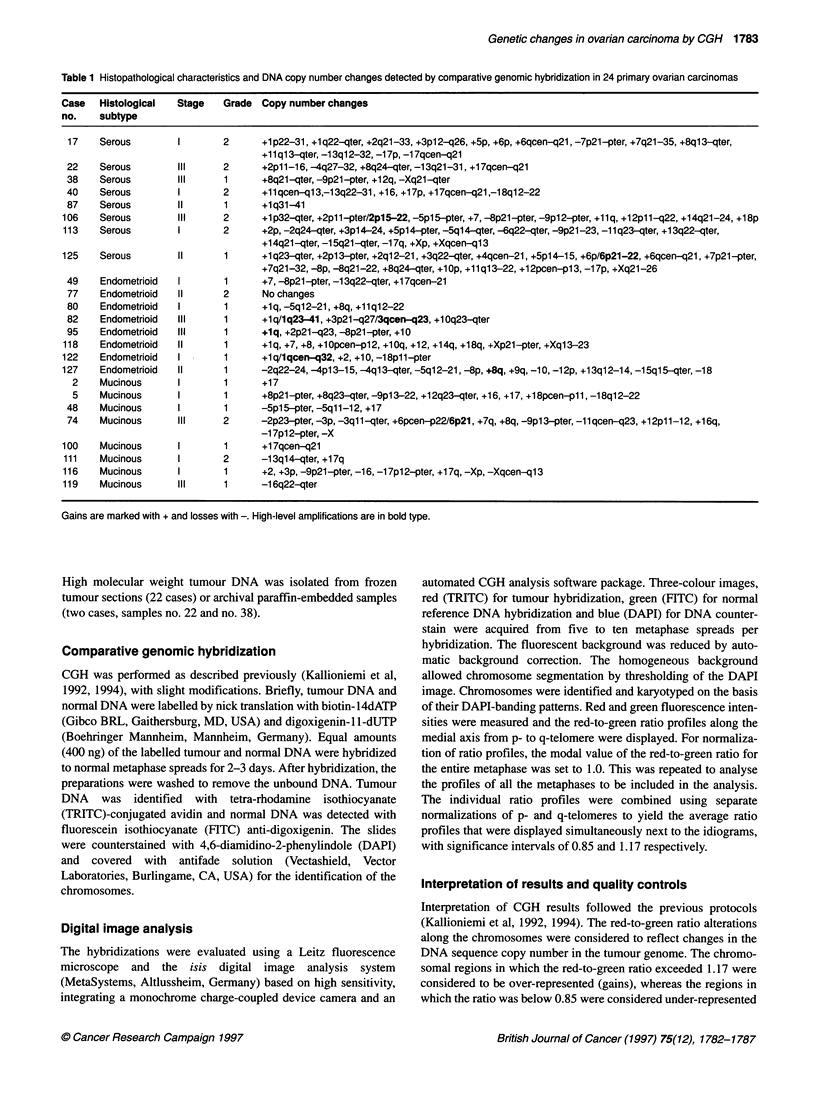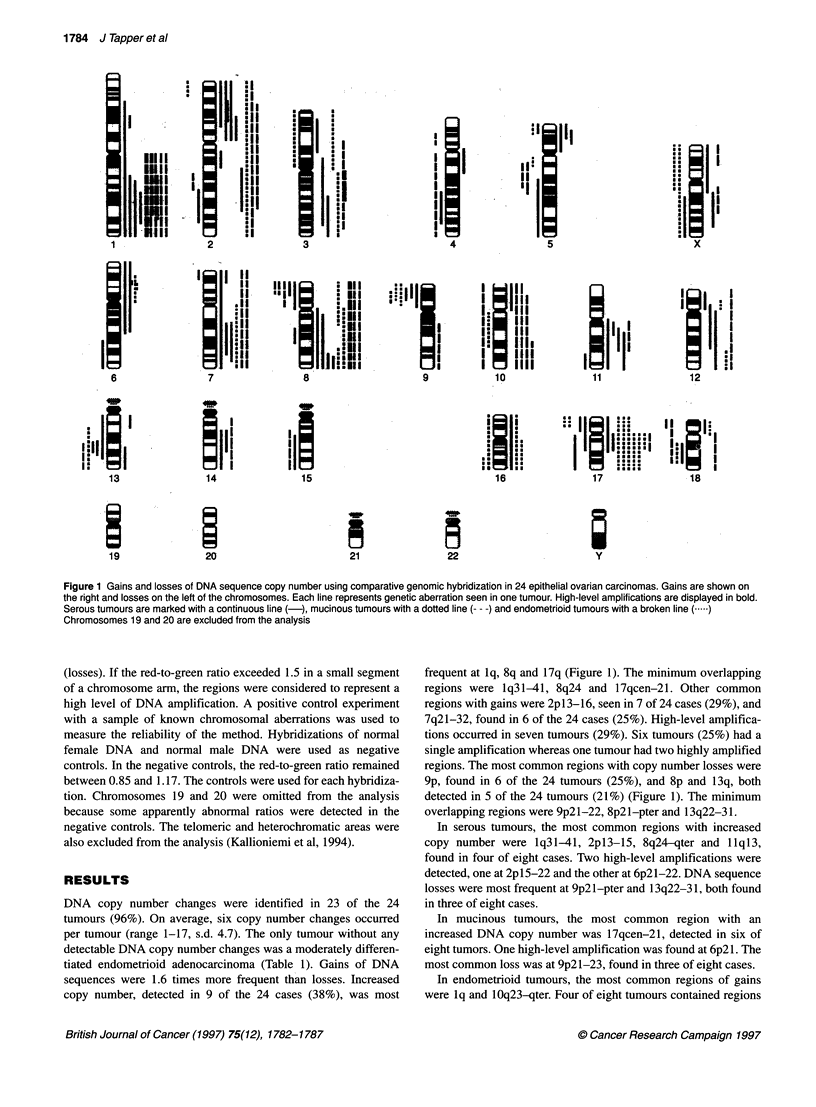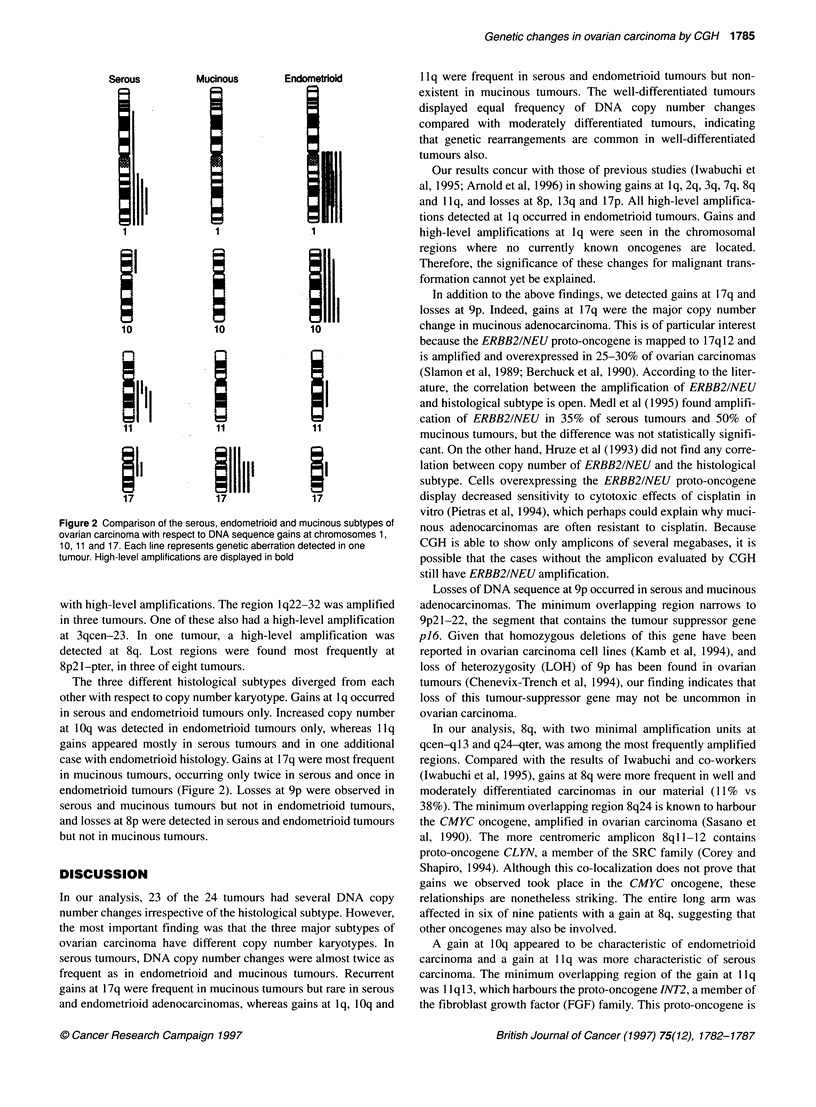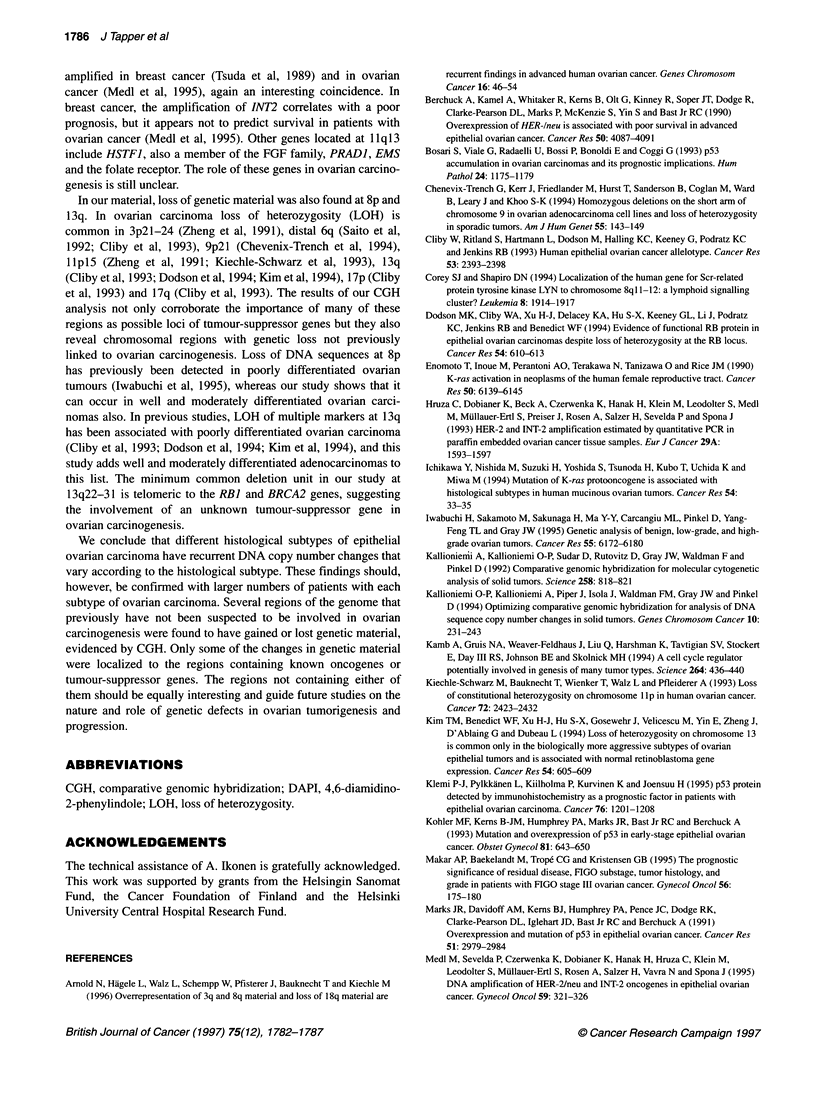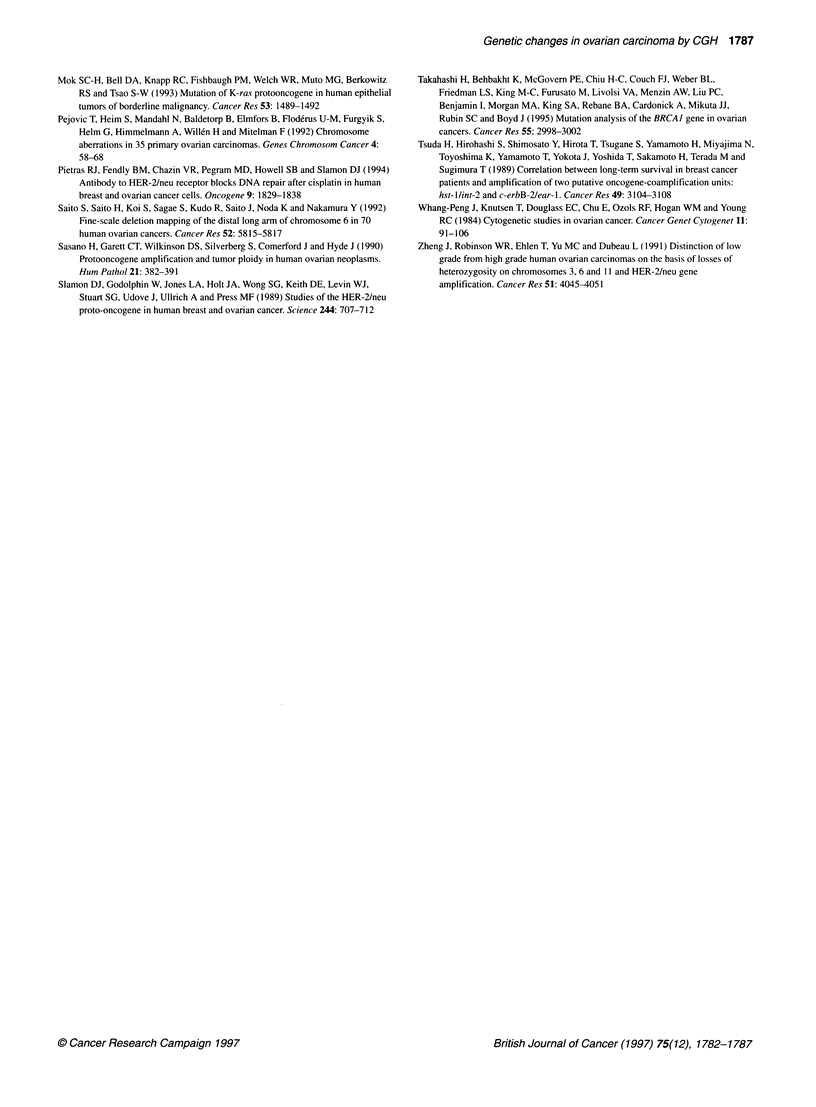# Evidence for divergence of DNA copy number changes in serous, mucinous and endometrioid ovarian carcinomas.

**DOI:** 10.1038/bjc.1997.304

**Published:** 1997

**Authors:** J. Tapper, R. BÃ¼tzow, T. WahlstrÃ¶m, M. SeppÃ¤lÃ¤, S. Knuutila

**Affiliations:** Department of Obstetrics and Gynecology, Helsinki University Central Hospital, Finland.

## Abstract

Comparative genomic hybridization was applied to detect and map changes in DNA copy number in 24 well or moderately differentiated epithelial ovarian carcinomas (eight serous, eight mucinous and eight endometrioid carcinomas). Twenty-three of the 24 tumours showed changes in DNA copy number in one or several regions (median 4, range 1-17). Gains were more frequent than losses (ratio 1.6:1.0). The most frequent gains occurred in chromosomes 1q (38%), 2p (29%), 7q (25%), 8q(38%) and 17q (38%), and the most common losses were located in chromosomes 8p (21%), 9p (25%) and 13q (21%). High-level amplifications were detected in seven tumours at 1q22-32, 2p15-22, 3qcen-23, 6p21-22 and 8q. In the three histological subtypes the copy number karyotypes showed substantial differences. Gains at 1q were observed in endometrioid (five cases) and serous tumours (four cases). Increased copy number at 10q was seen in endometrioid tumours only (four cases), whereas gains at 11q occurred mostly in serous tumours (four cases). In mucinous tumours, the most common copy number change was a gain at 17q (six cases). The results show that, in epithelial ovarian carcinoma, changes in DNA copy number are a rule rather than an exception, chromosomes 1, 2, 7, 8, 9, 13 and 17 being the most frequently affected. The diverging pattern of genetic changes seen in epithelial ovarian carcinomas with different histological phenotypes suggests that various pathways may lead to tumorigenesis and/or progression in these subgroups.


British Journal of Cancer (1997) 75(12), 1782–1787
© 1997 Cancer Research Campaign

### Evidence for divergence of DNA copy number changes in serous, mucinous and endometrioid ovarian carcinomas

**J Tapper[1], R Bützow[1], T Wahlström[1], M Seppälä[1] and S Knuutila[2]**

[1]Department of Obstetrics and Gynecology, Helsinki University Central Hospital, Haartmaninkatu 2, FIN-00290 Helsinki, Finland; [2]Department of Medical Genetics, Haartman Institute, POB 21 (Haartmaninkatu 3), FIN-00014 University of Helsinki, Helsinki, Finland

**Summary** Comparative genomic hybridization was applied to detect and map changes in DNA copy number in 24 well or moderately differentiated epithelial ovarian carcinomas (eight serous, eight mucinous and eight endometrioid carcinomas). Twenty-three of the 24 tumours showed changes in DNA copy number in one or several regions (median 4, range 1–17). Gains were more frequent than losses (ratio 1.6:1.0). The most frequent gains occurred in chromosomes 1q (38%), 2p (29%), 7q (25%), 8q(38%) and 17q (38%), and the most common losses were located in chromosomes 8p (21%), 9p (25%) and 13q (21%). High-level amplifications were detected in seven tumours at 1q22–32, 2p15–22, 3qcen–23, 6p21–22 and 8q. In the three histological subtypes the copy number karyotypes showed substantial differences. Gains at 1q were observed in endometrioid (five cases) and serous tumours (four cases). Increased copy number at 10q was seen in endometrioid tumours only (four cases), whereas gains at 11q occurred mostly in serous tumours (four cases). In mucinous tumours, the most common copy number change was a gain at 17q (six cases). The results show that, in epithelial ovarian carcinoma, changes in DNA copy number are a rule rather than an exception, chromosomes 1, 2, 7, 8, 9, 13 and 17 being the most frequently affected. The diverging pattern of genetic changes seen in epithelial ovarian carcinomas with different histological phenotypes suggests that various pathways may lead to tumorigenesis and/or progression in these subgroups.

**Keywords:** comparative genomic hybridization; ovarian carcinoma; DNA copy number change; histology

About 90% of ovarian cancers originate from the gonadal epithelium. The majority of ovarian carcinomas are histologically serous, mucinous or endometrioid, classified according to the epithelial component. It is not known whether these histological subtypes share common genetic pathways during tumorigenesis. Treatment of these different subtypes follows the same principles. The studies by Makar et al (1995) show that endometrioid tumours have the best prognosis whereas serous tumours make up the majority and carry an intermediate prognosis. Mucinous tumours have the poorest outcome, probably due to resistance to chemotherapy (Makar et al, 1995).

Complex genetic rearrangement is typical of moderately and poorly differentiated but not of well-differentiated ovarian carcinoma (Whang-Peng et al, 1984, Pejovic et al, 1992). Molecular genetic analyses of ovarian carcinoma have suggested a genotype–phenotype correlation. Mutation of the *KRAS* proto-oncogene is common, being more frequent in mucinous carcinoma than in serous carcinoma (Enomoto et al, 1990; Mok et al, 1993; Ichikawa et al, 1994). Of the known tumour-suppressor genes, the high incidence of *p53* gene point mutations, present in some 50% of these tumours, is found in epithelial ovarian carcinomas (Marks et al, 1991). Such mutations have also been reported to occur in more than half of the serous carcinomas, in about one-third of the endometrioid carcinomas, but not in the mucinous carcinomas (Klemi et al, 1995). However, this finding has not been seen in all studies (Bosari et al, 1993; Kohler et al, 1993). There seems to be a complete lack of mucinous subtype among *BRCA1*-associated hereditary ovarian carcinomas (Takahashi et al, 1995).

Karyotype analysis, allelotyping, Southern blotting and gene expression studies have been performed to identify oncogenes and tumour-suppressor genes involved in ovarian carcinogenesis. Recently, comparative genomic hybridization (CGH) has been developed to survey entire genomes for DNA sequence copy number variation (Kallioniemi et al, 1992). CGH enables screening for gains and losses of DNA sequences along all the chromosome arms. The method is based on the concept that chromosomal regions with an increased copy number reveal dominant oncogenes, whereas regions with a decreased copy number indicate putative tumour-suppressor gene loci (Kallioniemi et al, 1992). We used CGH to identify and map changes in DNA sequence copy number in the three most common histological subtypes of epithelial ovarian carcinoma to find out whether the different subtypes diverge with respect to the DNA copy number karyotype.

*Received 10 September 1996*
*Revised 4 December 1996*
*Accepted 12 December 1996*

*Correspondence to:* R Bützow

### MATERIAL AND METHODS

### Tumour specimens

The material consisted of 24 epithelial ovarian cancers: eight serous cystadenocarcinomas, eight endometrioid carcinomas and eight mucinous cystadenocarcinomas of various stages, diagnosed and treated at the Department of Obstetrics and Gynecology, Helsinki University Central Hospital, Helsinki, Finland (Table 1).

**Table 1** Histopathological characteristics and DNA copy number changes detected by comparative genomic hybridization in 24 primary ovarian carcinomas

| Case no. | Histological subtype | Stage | Grade | Copy number changes |
|---|---|---|---|---|
| 17 | Serous | I | 2 | +1p22–31, +1q22–qter, +2q21–33, +3p12–q26, +5p, +6p, +6qcen–q21, –7p21–pter, +7q21–35, +8q13–qter, +11q13–qter, –13q12–32, –17p, –17qcen–q21 |
| 22 | Serous | III | 2 | +2p11–16, –4q27–32, +8q24–qter, –13q21–31, +17qcen–q21 |
| 38 | Serous | III | 1 | +8q21–qter, –9p21–pter, +12q, –Xq21–qter |
| 40 | Serous | I | 2 | +11qcen–q13,–13q22–31, +16, +17p, +17qcen–q21,–18q12–22 |
| 87 | Serous | II | 1 | +1q31–41 |
| 106 | Serous | III | 2 | +1p32–qter, +2p11–pter/**2p15–22**, –5p15–pter, +7, –8p21–pter, –9p12–pter, +11q, +12p11–q22, +14q21–24, +18p |
| 113 | Serous | I | 2 | +2p, –2q24–qter, +3p14–24, +5p14–pter, –5q14–qter, –6q22–qter, –9p21–23, –11q23–qter, +13q22–qter, +14q21–qter, –15q21–qter, –17q, +Xp, +Xqcen–q13 |
| 125 | Serous | II | 1 | +1q23–qter, +2p13–pter, +2q12–21, +3q22–qter, +4qcen–21, +5p14–15, +6p/**6p21–22**, +6qcen–q21, +7p21–pter, +7q21–32, –8p, –8q21–22, +8q24–qter, +10p, +11q13–22, +12pcen–p13, –17p, +Xq21–26 |
| 49 | Endometrioid | I | 1 | +7, –8p21–pter, –13q22–qter, +17qcen–21 |
| 77 | Endometrioid | II | 2 | No changes |
| 80 | Endometrioid | I | 1 | +1q, –5q12–21, +8q, +11q12–22 |
| 82 | Endometrioid | III | 1 | +1q/**1q23–41**, +3p21–q27/**3qcen–q23**, +10q23–qter |
| 95 | Endometrioid | III | 1 | **+1q**, +2p21–q23, –8p21–pter, +10 |
| 118 | Endometrioid | II | 1 | +1q, +7, +8, +10pcen–p12, +10q, +12, +14q, +18q, +Xp21–pter, +Xq13–23 |
| 122 | Endometrioid | I | 1 | +1q/**1qcen–q32**, +2, +10, –18p11–pter |
| 127 | Endometrioid | II | 1 | –2q22–24, –4p13–15, –4q13–qter, –5q12–21, –8p, **+8q**, +9q, –10, –12p, +13q12–14, –15q15–qter, –18 |
| 2 | Mucinous | I | 1 | +17 |
| 5 | Mucinous | I | 1 | +8p21–pter, +8q23–qter, –9p13–22, +12q23–qter, +16, +17, +18pcen–p11, –18q12–22 |
| 48 | Mucinous | I | 1 | –5p15–pter, –5q11–12, +17 |
| 74 | Mucinous | III | 2 | –2p23–pter, –3p, –3q11–qter, +6pcen–p22/**6p21**, +7q, +8q, –9p13–pter, –11qcen–q23, +12p11–12, +16q, –17p12–pter, –X |
| 100 | Mucinous | I | 1 | +17qcen–q21 |
| 111 | Mucinous | I | 2 | –13q14–qter, +17q |
| 116 | Mucinous | I | 1 | +2, +3p, –9p21–pter, –16, –17p12–pter, +17q, –Xp, –Xqcen–q13 |
| 119 | Mucinous | III | 1 | –16q22–qter |

Gains are marked with + and losses with –. High-level amplifications are in bold type.

High molecular weight tumour DNA was isolated from frozen tumour sections (22 cases) or archival paraffin-embedded samples (two cases, samples no. 22 and no. 38).

### Comparative genomic hybridization

CGH was performed as described previously (Kallioniemi et al, 1992, 1994), with slight modifications. Briefly, tumour DNA and normal DNA were labelled by nick translation with biotin-14dATP (Gibco BRL, Gaithersburg, MD, USA) and digoxigenin-11-dUTP (Boehringer Mannheim, Mannheim, Germany). Equal amounts (400 ng) of the labelled tumour and normal DNA were hybridized to normal metaphase spreads for 2–3 days. After hybridization, the preparations were washed to remove the unbound DNA. Tumour DNA was identified with tetra-rhodamine isothiocyanate (TRITC)-conjugated avidin and normal DNA was detected with fluorescein isothiocyanate (FITC) anti-digoxigenin. The slides were counterstained with 4,6-diamidino-2-phenylindole (DAPI) and covered with antifade solution (Vectashield, Vector Laboratories, Burlingame, CA, USA) for the identification of the chromosomes.

### Digital image analysis

The hybridizations were evaluated using a Leitz fluorescence microscope and the *isis* digital image analysis system (MetaSystems, Altlussheim, Germany) based on high sensitivity, integrating a monochrome charge-coupled device camera and an automated CGH analysis software package. Three-colour images, red (TRITC) for tumour hybridization, green (FITC) for normal reference DNA hybridization and blue (DAPI) for DNA counterstain were acquired from five to ten metaphase spreads per hybridization. The fluorescent background was reduced by automatic background correction. The homogeneous background allowed chromosome segmentation by thresholding of the DAPI image. Chromosomes were identified and karyotyped on the basis of their DAPI-banding patterns. Red and green fluorescence intensities were measured and the red-to-green ratio profiles along the medial axis from p- to q-telomere were displayed. For normalization of ratio profiles, the modal value of the red-to-green ratio for the entire metaphase was set to 1.0. This was repeated to analyse the profiles of all the metaphases to be included in the analysis. The individual ratio profiles were combined using separate normalizations of p- and q-telomeres to yield the average ratio profiles that were displayed simultaneously next to the idiograms, with significance intervals of 0.85 and 1.17 respectively.

### Interpretation of results and quality controls

Interpretation of CGH results followed the previous protocols (Kallioniemi et al, 1992, 1994). The red-to-green ratio alterations along the chromosomes were considered to reflect changes in the DNA sequence copy number in the tumour genome. The chromosomal regions in which the red-to-green ratio exceeded 1.17 were considered to be over-represented (gains), whereas the regions in which the ratio was below 0.85 were considered under-represented

© Cancer Research Campaign 1997

**Figure 1** Gains and losses of DNA sequence copy number using comparative genomic hybridization in 24 epithelial ovarian carcinomas. Gains are shown on the right and losses on the left of the chromosomes. Each line represents genetic aberration seen in one tumour. High-level amplifications are displayed in bold. Serous tumours are marked with a continuous line (—), mucinous tumours with a dotted line (- - -) and endometrioid tumours with a broken line (·····) Chromosomes 19 and 20 are excluded from the analysis

(losses). If the red-to-green ratio exceeded 1.5 in a small segment of a chromosome arm, the regions were considered to represent a high level of DNA amplification. A positive control experiment with a sample of known chromosomal aberrations was used to measure the reliability of the method. Hybridizations of normal female DNA and normal male DNA were used as negative controls. In the negative controls, the red-to-green ratio remained between 0.85 and 1.17. The controls were used for each hybridization. Chromosomes 19 and 20 were omitted from the analysis because some apparently abnormal ratios were detected in the negative controls. The telomeric and heterochromatic areas were also excluded from the analysis (Kallioniemi et al, 1994).

### RESULTS

DNA copy number changes were identified in 23 of the 24 tumours (96%). On average, six copy number changes occurred per tumour (range 1–17, s.d. 4.7). The only tumour without any detectable DNA copy number changes was a moderately differentiated endometrioid adenocarcinoma (Table 1). Gains of DNA sequences were 1.6 times more frequent than losses. Increased copy number, detected in 9 of the 24 cases (38%), was most frequent at 1q, 8q and 17q (Figure 1). The minimum overlapping regions were 1q31–41, 8q24 and 17qcen–21. Other common regions with gains were 2p13–16, seen in 7 of 24 cases (29%), and 7q21–32, found in 6 of the 24 cases (25%). High-level amplifications occurred in seven tumours (29%). Six tumours (25%) had a single amplification whereas one tumour had two highly amplified regions. The most common regions with copy number losses were 9p, found in 6 of the 24 tumours (25%), and 8p and 13q, both detected in 5 of the 24 tumours (21%) (Figure 1). The minimum overlapping regions were 9p21–22, 8p21–pter and 13q22–31.

In serous tumours, the most common regions with increased copy number were 1q31–41, 2p13–15, 8q24–qter and 11q13, found in four of eight cases. Two high-level amplifications were detected, one at 2p15–22 and the other at 6p21–22. DNA sequence losses were most frequent at 9p21–pter and 13q22–31, both found in three of eight cases.

In mucinous tumours, the most common region with an increased DNA copy number was 17qcen–21, detected in six of eight tumors. One high-level amplification was found at 6p21. The most common loss was at 9p21–23, found in three of eight cases.

In endometrioid tumours, the most common regions of gains were 1q and 10q23–qter. Four of eight tumours contained regions

*© Cancer Research Campaign 1997*

**Figure 2** Comparison of the serous, endometrioid and mucinous subtypes of ovarian carcinoma with respect to DNA sequence gains at chromosomes 1, 10, 11 and 17. Each line represents genetic aberration detected in one tumour. High-level amplifications are displayed in bold with high-level amplifications. The region 1q22–32 was amplified in three tumours. One of these also had a high-level amplification at 3qcen–23. In one tumour, a high-level amplification was detected at 8q. Lost regions were found most frequently at 8p21–pter, in three of eight tumours.

The three different histological subtypes diverged from each other with respect to copy number karyotype. Gains at 1q occurred in serous and endometrioid tumours only. Increased copy number at 10q was detected in endometrioid tumours only, whereas 11q gains appeared mostly in serous tumours and in one additional case with endometrioid histology. Gains at 17q were most frequent in mucinous tumours, occurring only twice in serous and once in endometrioid tumours (Figure 2). Losses at 9p were observed in serous and mucinous tumours but not in endometrioid tumours, and losses at 8p were detected in serous and endometrioid tumours but not in mucinous tumours.

### DISCUSSION

In our analysis, 23 of the 24 tumours had several DNA copy number changes irrespective of the histological subtype. However, the most important finding was that the three major subtypes of ovarian carcinoma have different copy number karyotypes. In serous tumours, DNA copy number changes were almost twice as frequent as in endometrioid and mucinous tumours. Recurrent gains at 17q were frequent in mucinous tumours but rare in serous and endometrioid adenocarcinomas, whereas gains at 1q, 10q and 11q were frequent in serous and endometrioid tumours but non-existent in mucinous tumours. The well-differentiated tumours displayed equal frequency of DNA copy number changes compared with moderately differentiated tumours, indicating that genetic rearrangements are common in well-differentiated tumours also.

Our results concur with those of previous studies (Iwabuchi et al, 1995; Arnold et al, 1996) in showing gains at 1q, 2q, 3q, 7q, 8q and 11q, and losses at 8p, 13q and 17p. All high-level amplifications detected at 1q occurred in endometrioid tumours. Gains and high-level amplifications at 1q were seen in the chromosomal regions where no currently known oncogenes are located. Therefore, the significance of these changes for malignant transformation cannot yet be explained.

In addition to the above findings, we detected gains at 17q and losses at 9p. Indeed, gains at 17q were the major copy number change in mucinous adenocarcinoma. This is of particular interest because the *ERBB2/NEU* proto-oncogene is mapped to 17q12 and is amplified and overexpressed in 25–30% of ovarian carcinomas (Slamon et al, 1989; Berchuck et al, 1990). According to the literature, the correlation between the amplification of *ERBB2/NEU* and histological subtype is open. Medl et al (1995) found amplification of *ERBB2/NEU* in 35% of serous tumours and 50% of mucinous tumours, but the difference was not statistically significant. On the other hand, Hruze et al (1993) did not find any correlation between copy number of *ERBB2/NEU* and the histological subtype. Cells overexpressing the *ERBB2/NEU* proto-oncogene display decreased sensitivity to cytotoxic effects of cisplatin in vitro (Pietras et al, 1994), which perhaps could explain why mucinous adenocarcinomas are often resistant to cisplatin. Because CGH is able to show only amplicons of several megabases, it is possible that the cases without the amplicon evaluated by CGH still have *ERBB2/NEU* amplification.

Losses of DNA sequence at 9p occurred in serous and mucinous adenocarcinomas. The minimum overlapping region narrows to 9p21–22, the segment that contains the tumour suppressor gene *p16*. Given that homozygous deletions of this gene have been reported in ovarian carcinoma cell lines (Kamb et al, 1994), and loss of heterozygosity (LOH) of 9p has been found in ovarian tumours (Chenevix-Trench et al, 1994), our finding indicates that loss of this tumour-suppressor gene may not be uncommon in ovarian carcinoma.

In our analysis, 8q, with two minimal amplification units at qcen–q13 and q24–qter, was among the most frequently amplified regions. Compared with the results of Iwabuchi and co-workers (Iwabuchi et al, 1995), gains at 8q were more frequent in well and moderately differentiated carcinomas in our material (11% vs 38%). The minimum overlapping region 8q24 is known to harbour the *CMYC* oncogene, amplified in ovarian carcinoma (Sasano et al, 1990). The more centromeric amplicon 8q11–12 contains proto-oncogene *CLYN*, a member of the SRC family (Corey and Shapiro, 1994). Although this co-localization does not prove that gains we observed took place in the *CMYC* oncogene, these relationships are nonetheless striking. The entire long arm was affected in six of nine patients with a gain at 8q, suggesting that other oncogenes may also be involved.

A gain at 10q appeared to be characteristic of endometrioid carcinoma and a gain at 11q was more characteristic of serous carcinoma. The minimum overlapping region of the gain at 11q was 11q13, which harbours the proto-oncogene *INT2*, a member of the fibroblast growth factor (FGF) family. This proto-oncogene is

© Cancer Research Campaign 1997 

amplified in breast cancer (Tsuda et al, 1989) and in ovarian cancer (Medl et al, 1995), again an interesting coincidence. In breast cancer, the amplification of *INT2* correlates with a poor prognosis, but it appears not to predict survival in patients with ovarian cancer (Medl et al, 1995). Other genes located at 11q13 include *HSTF1*, also a member of the FGF family, *PRAD1*, *EMS* and the folate receptor. The role of these genes in ovarian carcinogenesis is still unclear.

In our material, loss of genetic material was also found at 8p and 13q. In ovarian carcinoma loss of heterozygosity (LOH) is common in 3p21–24 (Zheng et al, 1991), distal 6q (Saito et al, 1992; Cliby et al, 1993), 9p21 (Chevenix-Trench et al, 1994), 11p15 (Zheng et al, 1991; Kiechle-Schwarz et al, 1993), 13q (Cliby et al, 1993; Dodson et al, 1994; Kim et al, 1994), 17p (Cliby et al, 1993) and 17q (Cliby et al, 1993). The results of our CGH analysis not only corroborate the importance of many of these regions as possible loci of tumour-suppressor genes but they also reveal chromosomal regions with genetic loss not previously linked to ovarian carcinogenesis. Loss of DNA sequences at 8p has previously been detected in poorly differentiated ovarian tumours (Iwabuchi et al, 1995), whereas our study shows that it can occur in well and moderately differentiated ovarian carcinomas also. In previous studies, LOH of multiple markers at 13q has been associated with poorly differentiated ovarian carcinoma (Cliby et al, 1993; Dodson et al, 1994; Kim et al, 1994), and this study adds well and moderately differentiated adenocarcinomas to this list. The minimum common deletion unit in our study at 13q22–31 is telomeric to the *RB1* and *BRCA2* genes, suggesting the involvement of an unknown tumour-suppressor gene in ovarian carcinogenesis.

We conclude that different histological subtypes of epithelial ovarian carcinoma have recurrent DNA copy number changes that vary according to the histological subtype. These findings should, however, be confirmed with larger numbers of patients with each subtype of ovarian carcinoma. Several regions of the genome that previously have not been suspected to be involved in ovarian carcinogenesis were found to have gained or lost genetic material, evidenced by CGH. Only some of the changes in genetic material were localized to the regions containing known oncogenes or tumour-suppressor genes. The regions not containing either of them should be equally interesting and guide future studies on the nature and role of genetic defects in ovarian tumorigenesis and progression.

### ABBREVIATIONS

CGH, comparative genomic hybridization; DAPI, 4,6-diamidino-2-phenylindole; LOH, loss of heterozygosity.

### ACKNOWLEDGEMENTS

The technical assistance of A. Ikonen is gratefully acknowledged. This work was supported by grants from the Helsingin Sanomat Fund, the Cancer Foundation of Finland and the Helsinki University Central Hospital Research Fund.

### REFERENCES

Arnold N, Hägele L, Walz L, Schempp W, Pfisterer J, Bauknecht T and Kiechle M (1996) Overrepresentation of 3q and 8q material and loss of 18q material are recurrent findings in advanced human ovarian cancer. *Genes Chromosom Cancer* **16**: 46–54

Berchuck A, Kamel A, Whitaker R, Kerns B, Olt G, Kinney R, Soper JT, Dodge R, Clarke-Pearson DL, Marks P, McKenzie S, Yin S and Bast Jr RC (1990) Overexpression of *HER-/neu* is associated with poor survival in advanced epithelial ovarian cancer. *Cancer Res* **50**: 4087–4091

Bosari S, Viale G, Radaelli U, Bossi P, Bonoldi E and Coggi G (1993) p53 accumulation in ovarian carcinomas and its prognostic implications. *Hum Pathol* **24**: 1175–1179

Chenevix-Trench G, Kerr J, Friedlander M, Hurst T, Sanderson B, Coglan M, Ward B, Leary J and Khoo S-K (1994) Homozygous deletions on the short arm of chromosome 9 in ovarian adenocarcinoma cell lines and loss of heterozygosity in sporadic tumors. *Am J Hum Genet* **55**: 143–149

Cliby W, Ritland S, Hartmann L, Dodson M, Halling KC, Keeney G, Podratz KC and Jenkins RB (1993) Human epithelial ovarian cancer allelotype. *Cancer Res* **53**: 2393–2398

Corey SJ and Shapiro DN (1994) Localization of the human gene for Scr-related protein tyrosine kinase LYN to chromosome 8q11–12: a lymphoid signalling cluster? *Leukemia* **8**: 1914–1917

Dodson MK, Cliby WA, Xu H-J, Delacey KA, Hu S-X, Keeney GL, Li J, Podratz KC, Jenkins RB and Benedict WF (1994) Evidence of functional RB protein in epithelial ovarian carcinomas despite loss of heterozygosity at the RB locus. *Cancer Res* **54**: 610–613

Enomoto T, Inoue M, Perantoni AO, Terakawa N, Tanizawa O and Rice JM (1990) K-*ras* activation in neoplasms of the human female reproductive tract. *Cancer Res* **50**: 6139–6145

Hruza C, Dobianer K, Beck A, Czerwenka K, Hanak H, Klein M, Leodolter S, Medl M, Müllauer-Ertl S, Preiser J, Rosen A, Salzer H, Sevelda P and Spona J (1993) HER-2 and INT-2 amplification estimated by quantitative PCR in paraffin embedded ovarian cancer tissue samples. *Eur J Cancer* **29A**: 1593–1597

Ichikawa Y, Nishida M, Suzuki H, Yoshida S, Tsunoda H, Kubo T, Uchida K and Miwa M (1994) Mutation of K-*ras* protooncogene is associated with histological subtypes in human mucinous ovarian tumors. *Cancer Res* **54**: 33–35

Iwabuchi H, Sakamoto M, Sakunaga H, Ma Y-Y, Carcangiu ML, Pinkel D, Yang-Feng TL and Gray JW (1995) Genetic analysis of benign, low-grade, and high-grade ovarian tumors. *Cancer Res* **55**: 6172–6180

Kallioniemi A, Kallioniemi O-P, Sudar D, Rutovitz D, Gray JW, Waldman F and Pinkel D (1992) Comparative genomic hybridization for molecular cytogenetic analysis of solid tumors. *Science* **258**: 818–821

Kallioniemi O-P, Kallioniemi A, Piper J, Isola J, Waldman FM, Gray JW and Pinkel D (1994) Optimizing comparative genomic hybridization for analysis of DNA sequence copy number changes in solid tumors. *Genes Chromosom Cancer* **10**: 231–243

Kamb A, Gruis NA, Weaver-Feldhaus J, Liu Q, Harshman K, Tavtigian SV, Stockert E, Day III RS, Johnson BE and Skolnick MH (1994) A cell cycle regulator potentially involved in genesis of many tumor types. *Science* **264**: 436–440

Kiechle-Schwarz M, Bauknecht T, Wienker T, Walz L and Pfleiderer A (1993) Loss of constitutional heterozygosity on chromosome 11p in human ovarian cancer. *Cancer* **72**: 2423–2432

Kim TM, Benedict WF, Xu H-J, Hu S-X, Gosewehr J, Velicescu M, Yin E, Zheng J, D'Ablaing G and Dubeau L (1994) Loss of heterozygosity on chromosome 13 is common only in the biologically more aggressive subtypes of ovarian epithelial tumors and is associated with normal retinoblastoma gene expression. *Cancer Res* **54**: 605–609

Klemi P-J, Pylkkänen L, Kiilholma P, Kurvinen K and Joensuu H (1995) p53 protein detected by immunohistochemistry as a prognostic factor in patients with epithelial ovarian carcinoma. *Cancer* **76**: 1201–1208

Kohler MF, Kerns B-JM, Humphrey PA, Marks JR, Bast Jr RC and Berchuck A (1993) Mutation and overexpression of p53 in early-stage epithelial ovarian cancer. *Obstet Gynecol* **81**: 643–650

Makar AP, Baekelandt M, Tropé CG and Kristensen GB (1995) The prognostic significance of residual disease, FIGO substage, tumor histology, and grade in patients with FIGO stage III ovarian cancer. *Gynecol Oncol* **56**: 175–180

Marks JR, Davidoff AM, Kerns BJ, Humphrey PA, Pence JC, Dodge RK, Clarke-Pearson DL, Iglehart JD, Bast Jr RC and Berchuck A (1991) Overexpression and mutation of p53 in epithelial ovarian cancer. *Cancer Res* **51**: 2979–2984

Medl M, Sevelda P, Czerwenka K, Dobianer K, Hanak H, Hruza C, Klein M, Leodolter S, Müllauer-Ertl S, Rosen A, Salzer H, Vavra N and Spona J (1995) DNA amplification of HER-2/neu and INT-2 oncogenes in epithelial ovarian cancer. *Gynecol Oncol* **59**: 321–326

 © Cancer Research Campaign 1997

Mok SC-H, Bell DA, Knapp RC, Fishbaugh PM, Welch WR, Muto MG, Berkowitz RS and Tsao S-W (1993) Mutation of K-*ras* protooncogene in human epithelial tumors of borderline malignancy. *Cancer Res* **53**: 1489–1492

Pejovic T, Heim S, Mandahl N, Baldetorp B, Elmfors B, Flodérus U-M, Furgyik S, Helm G, Himmelmann A, Willén H and Mitelman F (1992) Chromosome aberrations in 35 primary ovarian carcinomas. *Genes Chromosom Cancer* **4**: 58–68

Pietras RJ, Fendly BM, Chazin VR, Pegram MD, Howell SB and Slamon DJ (1994) Antibody to HER-2/neu receptor blocks DNA repair after cisplatin in human breast and ovarian cancer cells. *Oncogene* **9**: 1829–1838

Saito S, Saito H, Koi S, Sagae S, Kudo R, Saito J, Noda K and Nakamura Y (1992) Fine-scale deletion mapping of the distal long arm of chromosome 6 in 70 human ovarian cancers. *Cancer Res* **52**: 5815–5817

Sasano H, Garett CT, Wilkinson DS, Silverberg S, Comerford J and Hyde J (1990) Protooncogene amplification and tumor ploidy in human ovarian neoplasms. *Hum Pathol* **21**: 382–391

Slamon DJ, Godolphin W, Jones LA, Holt JA, Wong SG, Keith DE, Levin WJ, Stuart SG, Udove J, Ullrich A and Press MF (1989) Studies of the HER-2/neu proto-oncogene in human breast and ovarian cancer. *Science* **244**: 707–712

Takahashi H, Behbakht K, McGovern PE, Chiu H-C, Couch FJ, Weber BL, Friedman LS, King M-C, Furusato M, Livolsi VA, Menzin AW, Liu PC, Benjamin I, Morgan MA, King SA, Rebane BA, Cardonick A, Mikuta JJ, Rubin SC and Boyd J (1995) Mutation analysis of the *BRCA1* gene in ovarian cancers. *Cancer Res* **55**: 2998–3002

Tsuda H, Hirohashi S, Shimosato Y, Hirota T, Tsugane S, Yamamoto H, Miyajima N, Toyoshima K, Yamamoto T, Yokota J, Yoshida T, Sakamoto H, Terada M and Sugimura T (1989) Correlation between long-term survival in breast cancer patients and amplification of two putative oncogene-coamplification units: *hst*-1/*int*-2 and *c-erb*B-2/*ear*-1. *Cancer Res* **49**: 3104–3108

Whang-Peng J, Knutsen T, Douglass EC, Chu E, Ozols RF, Hogan WM and Young RC (1984) Cytogenetic studies in ovarian cancer. *Cancer Genet Cytogenet* **11**: 91–106

Zheng J, Robinson WR, Ehlen T, Yu MC and Dubeau L (1991) Distinction of low grade from high grade human ovarian carcinomas on the basis of losses of heterozygosity on chromosomes 3, 6 and 11 and HER-2/neu gene amplification. *Cancer Res* **51**: 4045–4051

© Cancer Research Campaign 1997